# Nutrition-Related Practices and Attitudes of Kansas Skipped-Generation(s) Caregivers and Their Grandchildren

**DOI:** 10.3390/nu2121188

**Published:** 2010-11-30

**Authors:** Mary Meck Higgins, Bethany J. Murray

**Affiliations:** 1 Department of Human Nutrition, Kansas State University, Justin Hall, Manhattan, KS 66506, USA; 2 Department of Nutrition and Health Sciences, University of Nebraska-Lincoln, HECO Building, Lincoln, NE 68583, USA; Email: bethanymurray@att.net

**Keywords:** grandparents raising grandchildren, grandfamilies, grandparent caregivers, custodial grandparents, child nutrition, qualitative research, nutrition education, nutrition attitudes, food safety

## Abstract

Despite growing numbers, the nutrition practices and attitudes of skipped–generation(s) kinship caregivers regarding feeding the dependent children in their care have not been examined. In this qualitative study, transcriptions of semi-structured interviews with 19 female and four male skipped-generation(s) Kansas caregivers (ages 47 to 80, 92% non-Hispanic whites, 83% female, 78% grandparents and 22% great-aunt or great–grandparent caregivers; caring for a range of one to four children, ages three to 18, for an average of nine years) were content analyzed for how their nutrition-related practices and attitudes had changed since parenting the first time. Sub-themes regarding practices included: being more nutrition and food safety conscious now, and shifting their child feeding style. The children seemed to be adversely affected by an on-the-go lifestyle and the use of more electronics. Caregivers described their sources for child feeding advice as being based mostly on information from their mothers, physicians, and their past parenting experiences. Sub-themes for attitudes included opinions that nutrition and safe food handling are important and that nutritious food is expensive. They preferred printed or video nutrition education materials and wanted to receive information through organizations they trusted. This population could benefit from education on: infant, child, adolescent, and sports nutrition; feeding “picky eaters”; healthful recipes, “fast foods” and packaged foods; quick, inexpensive meals and snacks low in fat, sugar, and salt; limiting sedentary time; family meals; using food thermometers; and intergenerational gardening and cooking.

## 1. Introduction

The proportion of grandparents, or another older adult relative raising children, is increasing in the United States (U.S.). While many terms are used to describe this population, and we use them here interchangeably, “grandparent caregivers” are defined by the U.S. Census Bureau as “people who had primary responsibility for their co-resident grandchildren younger than 18”; they numbered 2,617,580 in 2008 [1]. The lifetime prevalence of grandparents raising grandchildren is higher than that reported in any year’s census data: grandparent caregiving that lasted for at least six months occurred for one in ten (10.9 percent) grandparents, based on 1992–1994 national data [2]. This percentage can be expected to be higher now, however, since the situation has become more common. 

The number of Kansan children who resided in a grandparent-headed household increased 43 percent between 1990 and 2000, which was 13 percent higher than the national average [3]. Approximately 19,995 grandparents were responsible for their grandchild(ren) in Kansas in 2008 [1]. Not only is the trend of skipped generation households increasing, the commitment generally lasts for an extended time. Among Kansan grandparents responsible for their grandchildren, 22 percent had cared for them one to two years, while 46 percent had shouldered this responsibility for three or more years [4]. 

Grandparent and other skipped-generation(s) kinship caregivers who provide parental care in the absence of the children’s biological parents experience unique physical, financial, familial, social, emotional, psychological and legal challenges. The implications of these have been well-published [5,6,7]. A collaboration of national organizations provides state-specific data and information about the range of support services, benefits, and policies that grandfamilies may need to fulfill caregiving roles [8]. One concern for grandparent caregivers is health problems for themselves—including high rates of depression, poor self-rated health, and the frequent presence of multiple chronic health problems—and their grandchildren [2]. Another concern is financial hardship. Grandparent caregivers are more likely than “all families with related children under 18 years of age” to have incomes below the poverty level, with 19 percent meeting this criterion in 2008 [1].

Eating a nourishing diet is yet another challenge faced by many people in this population. Of all older adults with limited resources, grandparents with responsibility for their grandchildren are the most likely to have low or very low food security [9]. Marginal food insecurity occurred in 30 percent of senior households with a grandchild but no adult child present, compared to 10 percent of households without a grandchild present. In a follow-up study, it was reported [10] that, nationally, 15.4 percent of senior households with a grandchild, but no adult child, present were at-risk of hunger, or food insecure, compared to 5.2 percent of households without a grandchild present. In Kansas, the rate of food insecurity was higher than the national average, at 16.8 percent, for families with a grandchild, but no adult child, present, compared to 4.6 percent for senior households without a grandchild present. Related to these findings, many grandparents with insufficient financial resources who are raising their grandchildren were found to decrease their grandchildren’s food portion sizes at times [11].

The interaction between generations in a household can affect the eating behaviors of both. For example, the presence of grandchildren influences grandparent caregivers’ dietary intakes. The three main perceived influences on healthful eating and physical activity of 18 urban, mostly female (marital status not reported), African-American grandparent caregivers were the presence of the grandchildren, cultural foods, and financial issues [12]. Grandchildren in the home negatively influenced these grandparents from making healthful dietary changes, stemming from consideration of their grandchildren’s tastes and food preferences, based on content analysis of the interviews. Also, cultural influences on diet and preferences for traditional foods, as well as the higher cost, were stated barriers to the caregivers eating more healthful diets.

Similarly, familial practices and attitudes can have a significant impact on the children’s dietary choices and psychosocial health [13,14]. For example, studies have shown that child feeding styles of parents that are either too controlling or too relaxed with child feeding can result in less healthful child dietary choices and weight status [15,16]. In one study of multiple generation households, both parents and grandparents expressed concern over their children and grandchildren’s unhealthful eating practices, and used multiple strategies to get family members to eat healthfully, but there was a lack of communication between all three generations at times concerning healthful nutrition behaviors [17].

The few published studies to date regarding nutrition and grandparents raising grandchildren have focused on education. For instance, in a report advising family service workers on how to assist grandparent caregivers, one team recommended working with them to develop a list of topics they should discuss with their health care provider, including: getting information about managing the child(ren)’s weight, nutrition, and fitness level; discussing the challenges of trying to meet their grandchild’s nutritional needs, and specifics related to meal planning, food budgets, and exercise options; and ensuring that their own specific health care needs were being met [18]. Only three research reports were found, and all showed that nutrition education, provided in conjunction with overall wellness or physical activity programs, increased the nutrition knowledge of grandparents raising their grandchildren [12,19,20].

Despite the rise in numbers of skipped-generation(s) households, the high prevalence of household food insecurity for custodial grandparents, and the interaction on eating behaviors between generations, the authors are not aware of any published research exploring nutrition-related practices or attitudes of skipped-generation(s) kinship caregivers living in the U.S. with regard to feeding their dependent children. Nutrition-related attitudes are opinions about particular eating, physical activity or food safety behaviors, and are associated with the adoption and maintenance of nutrition-related healthful habits [21,22]. Nutrition attitudes of individuals do not always correlate well with their reported nutrition practices, however [23,24]. Nutrition-related attitudes measure dietary preferences, perceptions about the role of food, and a person’s views about the benefits and feasibility of adopting healthier habits [23,25]. These attitudes can be positive (such as “a low-fat diet is enjoyable”) or negative (such as “eating healthfully is too much effort”) [26]. 

The purpose of our qualitative study was to investigate and uncover previously unarticulated nutrition-related practices and attitudes of grandparents or other skipped-generation(s) kinship caregivers and the dependent children in their care. Our goal for this report is to discuss what they perceived to be relevant issues related to adequately feeding the children for whom they were responsible, and in particular, how these nutrition-related practices and attitudes had changed from when they were parenting the first time.

## 2. Experimental Section

### 2.1. Design

The interviewer used a semi-structured interview guide format [27]. In-depth, face-to-face qualitative interviewing was the method of choice for this study for several reasons: the exploratory nature of the research (guided conversations with open-ended questions elicited stories from the participants about their experiences with a variety of nutrition practices and their attitudes, in their own words), the sensitive nature of the research topic (interviews were typically conducted in their own homes), and the convenience to the caregivers (not having to write down their opinions reduced the burden associated with written surveys, and they did not have to travel anywhere or arrange for alternative child care). 

### 2.2. Instrument Development

Although the interview guide (see Table 1) had six categories of questions, only the data from four portions are used in this report, reflecting the objectives regarding: nutrition-related attitudes, nutrition education, changes in their nutrition-related practices as second-time parents, and participant characteristics. 

**Table 1:** Main questions of the interview guide.

Categories	Main questions (Probes were used to elicit more information, as needed)
Participant background information	Can you briefly describe the children for whom you are primarily responsible? What advice would you offer to other grandparent caregivers? What have been the biggest challenges to you as a grandparent caregiver? Briefly, what were the circumstances behind your becoming the primary caregiver of the child(ren)?
Nutrition-related attitudes	What roles do food play in the child(ren)’s life? Do you have any concerns about the child(ren)’s eating habits? What would you say are the main things that influence your food shopping decisions when you are buying food?
Nutrition education	What were the most helpful sources of nutrition advice when you parented the first time? What are the most helpful sources of nutrition advice now that you are parenting for the second time? What kinds of nutrition education have been helpful in the past or are currently helpful? Are there any nutrition-related topics you would have liked to have had information on in the past? Are there any nutrition-related topics you would like information on now or for the future? Do you think grandparents would use nutrition-education materials designed specifically for them? What kinds of materials would be helpful? Is there anything else you would like me to know?
Nutrition-related practices	What does your grandchild eat on a typical day? Where does the child typically eat? Where are the food, snacks, and beverages for you and the child(ren) obtained or purchased? Do have a Vision [aka Supplemental Nutrition Assistance Program, or SNAP] card or other assistance to help with the children? What are some differences in some of the practices we just talked about concerning feeding your grandchild(ren) compared to what you did when you parented the first time? If the children spend time with their biological parents, how do feeding practices that we just talked about differ? What are some of the child(ren)’s favorite things to do? What kinds of physical activity do the child(ren) participate in? What kinds of things do you do to try to make sure your food is safe to eat?
Participant characteristics	Age, marital status, number of adults and children in household? Racial group you most identify with? Do you consider yourself Hispanic or Latino? Observed interviewee information included gender.

The guide was developed by the authors and tested with a family systems specialist who was also a grandmother living with, but not responsible for, her grandchild. During instrument development, questions were checked for singularity and clarity, as recommended by Patton [28]. 

### 2.3. Participants

To participate in the study, caregivers had to be (a) 45 years of age or older, (b) living in Kansas, (c) able to speak English, and (d) previously a parent and now the sole or primary caregiver for their (great)grandchild(ren) or another skipped-generation(s) child relative under the age of 18 years, where neither biological parent was present. This study used a purposeful snowball sampling strategy [29] to find information-rich key informants who met the participant criteria. Participants were recruited throughout the state through local grandparent support groups and referrals from K-State Research and Extension county faculty. Participant recruitment ended when no new sub-themes were being identified in new interviews.

### 2.4. Procedures

The study (number 4582) was approved on 3 March 2008 by the Kansas State University Committee on Research Involving Human Subjects. The interviewer contacted potential participants in person or via telephone to explain the study and answer questions. If they were interested, she then scheduled the in–person interview time. The face-to-face setting for each interview was selected by the participant. Participants were fully briefed and signed consent forms before the interviews. All interviews were recorded on audiotape with participants’ permission. Participants were told at the onset of the interviews that they could refuse to answer any question without penalty. Regardless of the number or depth of questions answered, participants were offered a $15 per family incentive. All oral interviews were conducted, using the interview guide, during a two-month period by one master’s level Registered Dietitian (second author, BJM), who had completed coursework and self-study in qualitative interviewing and research. The main questions were primarily open-ended. The protocol included the use of clarifying questions and follow-up probes designed to elicit details.

### 2.5. Data Analysis

Interviews were transcribed verbatim, using a word processor. The interviewer listening to the tapes while comparing them to the transcripts verified accuracy of the transcripts. Data analysis was carried out manually to find themes and sub-themes, both within each interview and between interviews. 

Principles described by Patton [30] guided the data analysis. All transcriptions were read several times to become fully familiar with the data. Transcribed quotes considered to represent the same concept were clustered together categorically, according to the researchers’ primary questions within the interview guide and additional unanticipated categories. The categorized quotes were then coded according to key phrases. We looked for patterns and repetition of key phrases, which we then sorted and re-sorted according to content similarities, to allow the “emergence” of sub-themes of data within our main predetermined thematic scheme in line with the interview guide topics. Peer debriefing was used throughout the study for increased credibility.

Descriptive statistics were obtained for demographic variables and screen time using Microsoft Excel (Microsoft Office, 2003).

## 3. Results

### 3.1. Participants

Participant characteristics are shown in Table 2. All caregivers appeared to be comfortable discussing the questions, based on their responses and body language; interviews averaged one hour but ranged from a half hour to two hours in length. Twenty-three caregivers representing 19 households and living in 17 counties across Kansas were interviewed (four male and 19 female). Caregivers ranged in age from 47 to 80 years, with a mean of 62 years. The majority of the caregivers were married (70%) non-Hispanic whites (92%). Length of care ranged from less than a year to 18 years, with a mean of nine years. The majority (78%) of those interviewed were grandparent caregivers. The remaining 22% were either great-aunt or great-grandparent caregivers. Thirty-seven percent of the households reported that they currently received some form of governmental monetary assistance. Only 11% of households lived in urban areas (>150 residents per square mile, or rpsm), with the remainder living in very rural or frontier areas of Kansas (37% in frontier areas, <6.0 rpsm; 26% in rural areas, 6.0–19.9 rpsm; and 26% in densely-settled rural areas, 20.0–39.9 rpsm).

**Table 2:** Participant characteristics.

Characteristic	n	Percentage	Average	Range
**Caregiver**				
Age, yrs	23		62	47–80
Married	16	70%		
Female	19	83%		
White, non-Hispanic	21	92%		
White, Hispanic	1	4%		
Native American	1	4%		
Length of care, yrs			9	<1–18
Relationship to child(ren):				
Grandparent	18	78%		
Great-grandparent	3	13%		
Great-aunt	2	9%		
Number of skipped-generation children for which the caregiver was primarily responsible			1	1–4
**Child**				
Age, yrs	25		12	3–18
Female	8	32%		
White, non-Hispanic	14	56%		
White, Hispanic	3	12%		
Mixed race/ethnicity	8	32%		
**Household Membership**	19			
Total people			3	2–10
Adults			2	1–3
Children			2	1–7
Receiving governmental assistance for:				
Food or other needs, or both	7	37%		
Medical only	2	10%		
Population density of residence				
Urban: >150.0 residents per square mile (rpsm)	2	11%		
Semi-urban: 40.0–149.9 rpsm	0	0%		
Densely-settled rural: 20.0–39.9 rpsm	5	26%		
Rural: 6.0–19.9 rpsm	5	26%		
Frontier: <6.0 rpsm	7	37%		

Their dependent child relatives (whom we will also refer to as “grandchildren”) were primarily non–Hispanic whites (56%) or of mixed descent (32%) and ranged in age from three to 18 years, with a mean of 12 years. Thirty-two percent were female. The average number of skipped-generation(s) children for which the caregivers were primarily responsible was one child, with a range from one to four children. The circumstances leading to the skipped-generation(s) care stemmed mostly from deemed parental inability because of child abuse or neglect, illegal activity, teen pregnancy, or mental health issues. Only three households (16%) reported they were providing care because of parental death. 

### 3.2. Themes

The nutrition-related findings from this study of skipped-generation(s) households were divided into two over-arching themes reflecting the objectives of the study: nutrition-related practices and nutrition–related attitudes. Several sub-themes emerged from each theme, as illustrated with quotes that convey how the participants described their practices and attitudes. Caregivers had a wide variety of nutrition-related practices and attitudes.

#### 3.2.1. Nutrition-Related Practices

Within the nutrition-related practices theme, five sub-themes were identified from the interview data. Compared to when they were parenting the first time, skipped-generation(s) caregivers overall reported that they are more nutrition and food safety conscious and that their grandchildren are adversely affected by an on-the-go lifestyle and the use of more electronics. Caregivers have shifted their child feeding style. Their sources of child feeding advice are based mostly on information from their mothers, physicians, and their past parenting experiences. 

##### 3.2.1.1. More nutrition and food safety conscious

Caregivers described practices that showed they are more nutrition conscious with raising their second generation of children than their first. The most common of these practices included providing a more nutritious variety of foods in the diet, including increasing servings of fruits, vegetables, and milk. Children in the majority of households ate diets that included a variety of foods from all food groups.

One food that I use more of with [my grandson] than I did with my kids: broccoli and cauliflower and more of the exotic fruits…. We’re more apt to try kiwi fruit or try more things. Sometimes [he] will see something and ask about it, and if we can afford it, we’ll get one and try it.

(grandmother 11)

In addition, seven households reported cooking foods from “scratch” more often now rather than from packages. Five households reported no change in this aspect of cooking. While five households described using more packaged foods now than with their first set of children, nevertheless all but a few of the caregivers who cook with more convenience foods also described eating more healthfully now than with their first set of children.

When we were first starting out, money was tighter, so when we shopped it was always the bargains. And sometimes the bargains weren’t always the healthiest choices…. They’re [packaged foods] always a bargain.... [Now] we want to eat healthier and want to eat the right way.

(grandmother 20)

Hamburger Helper used to be a staple when our children were young and we were learning how to cook. But we don’t even do that [now] because there’s so much sodium and stuff.

(grandmother 1)

Fewer packaged foods, I do more cooking [now]…. It was working and [taking care of] three [kids], and now it’s not working and [taking care of] one.

(grandmother 13)

I used to make all my cakes from scratch. I don’t make cakes much anymore, but when I do, I use a box [mix].

(grandmother 4)

[I use more packaged food now,] yes, it’s quicker. I used to cook pretty much from scratch. We do eat a lot of vegetables…. It was a bad habit to bake, but we don’t need all of that, so I quit. And we always have a dessert of some sort, but mostly fruit.

(grandmother 3)

Participants also described reading the Nutrition Facts labels on packaged products: 13 households did and five did not use them. Fat, sugar, and sodium content were the items on the label they looked at most often.

I don’t remember people being as nutrition-conscious or reading labels then…. Now I pay a lot more attention to them…. I’ve tried to be more conscious of what I feed him [my grandson]… [and] cook a lot of stuff from scratch.

(grandmother 10)

Being the age I am, I can kind of tell what’s good and everything. But yeah, we’re real aware of what’s inside of a package and [my grandson] will read them [Nutrition Facts labels] off. And so (laughs), that’s one of the first things he does, is flips something over and says, “Do you know how much sodium is in here?”… Yeah, so we read a lot of labels.

(grandmother 4)

I’ve been raising kids for 30 years and I really have not [used the Nutrition Facts labels]. I guess I just kind of depend on what I’ve done in the past.

(grandmother 15)

In addition to their nutrition-related behaviors, participants described increasing many practices with food safety in mind. They mentioned a wide variety of actions, including checking expiration dates on food or leftovers, paying attention to food recalls, refrigerating or freezing perishable foods promptly after shopping and after eating meals, checking refrigerator temperatures, washing their hands, washing fresh produce, and keeping food areas clean during preparation. Although six of the households reported having a food thermometer, only one caregiver reported using it when cooking.

We do the expiration date thing…. We buy food that doesn’t sit for long periods of time. That way it keeps it from going bad or not as nutritional further down the road.

(grandmother 1)

Well, I make sure that the refrigerator’s on the right temperature. I make sure things are covered and put in the refrigerator. I make sure that the meat is cooked. I’m very conscious about that. I make sure that the raw vegetables are washed. I’m pretty conscious about food safety. And I teach the kids that if you want some fruit, you wash it first.

(grandmother 7)

Caregivers commonly attributed their improved awareness to having: more time to plan meals and cook, more information about nutrition and food safety because it was more available and emphasized more in modern society, and, in some cases, more financial resources.

You don’t have time or pay attention to what they’re [your children] eating as working parents, because you want to get them fed as quickly as you possibly can. My focus entirely changed when I became a stay at home [grand]parent.

(grandfather 2)

Well, we didn’t have very many dollars. I’ve got more dollars now, and just one kid.

(great-grandmother 14)

##### 3.2.1.2. On-the-go lifestyle reduces healthful eating

Although caregivers reported many healthful changes in raising their second set of children, such as planning nutritious meals, these skipped-generation(s) households as a whole reported some less healthful changes because of the shift in society toward a busy, on-the-go lifestyle. Even though the caregivers and their grandchildren were more aware of nutrition, that knowledge at times did not result in the most healthful food selections. Some grandparents noted specific occasions for the increased availability and consumption of packaged products, pizza, “junk” foods, and “fast food” by the children in their care.

Every once in a while I try to tell him [my grandson] he’s eating too much junk but it doesn’t do no good…. I think he eats too much junk food, like McDonald’s every day…. ‘Cause he eats lunch out. The boys [my sons] didn’t have any place to go, they had to eat at school, and our daughter had to eat lunch there. We didn’t have McDonalds or Sonic or any of those things.

(grandfather 9)

We probably do more frozen pizzas occasionally ‘cause she [my great-niece] likes to get them. I do make them from scratch sometimes, too. If they have them on sale, she’ll say, “Let’s get a pizza.” And they’re nice too, because you can pull them out and they’re fast.

(great-aunt 23)

Some caregivers attributed their grandchildren’s occasional poor food choices to having less time to eat healthfully and needing to eat something quick for breakfast or “fast food” when they were away from home. Fourteen of the 19 households had at least one child involved in team sports, which took a lot of time and was often coupled with selecting quick, less healthful foods. The households with teenage boys with driver’s licenses seemed to eat the least healthfully. Because of the irregularity of these boys’ schedules, they had an almost daily consumption of “fast foods” and high amounts of “junk” snack foods.

They [our grandsons] really don’t want to sit down for a good meal. All they want is junk food. Our kids didn’t really eat junk food. It seems like they’re [our grandsons] not here half the time.

(grandfather 19)

Caregivers in four households specifically mentioned that they used to grow their own vegetables, and some even produced their own meat, eggs, milk, and fruits, but now they buy them.

We had a big garden… so I didn’t buy a lot of stuff. I canned a lot.

(grandmother 3)

##### 3.2.1.3. More electronics increase sedentary activities and purchases of advertised foods

Caregivers noted the increase between generations in children’s use of electronics, from hand-held video games to computers. In discussing the child(ren)’s favorite things to do, everyone described screen time as one of these activities. Responses regarding how much time was spent ranged from one caregiver reporting “very seldom”, to several saying “a lot”. Six households were unable to estimate specific amounts of time, and these were evenly split between “lots” and “a little”. Thirteen households gave estimates of the specific range of time that the children spent with computers, videos, and television: one to four hours each day. The authors averaged these responses for the group, and concluded that the children spent at least 2.5 hours a day with electronics. Several caregivers described actively monitoring and limiting time spent, while others spoke of their grandchildren spending “too much time” in sedentary activities: watching television, playing video games, and playing on the computer.

He [my grandson] loves video games, TV, computer. But more than the strenuous activity, he’s more of a computer guy…. I’d say most of the day, until I get home and then I boot him out. So, quite a bit actually. If I didn’t work it would be different, but I’m not there to get him off.

(grandmother 21)

Our kids never had video games, but as far as the rest of it [child rearing between generations], it’s pretty much the same.

(grandmother 18)

Another thing is, my kids didn’t have all the electronic stuff. I’m not for it…. I pay attention to what games he [my grandson] plays.

(grandmother 22)

In addition, the use of electronics was described as affecting the types of food purchased in some households. After the youngsters watched advertisements, such as on television, they sometimes asked their caregivers to purchase those specific foods.

The food he [my grandson] wanted went with the cartoon characters…. You had to have cereal. “Oh, they’ve got stuff in here. They’ve got all kinds of toys in this cereal, Granny.”

(grandmother 12)

The kids have say-so, you know, things they see on TV. I think a lot [of what we buy] is influenced by the kids, what they want to eat. Basically, we don’t always buy it, but he’s [my grandson] pretty influenced, since he watches quite a bit of TV.

(grandmother 21)

… Like that V-8 Fusion in that drink. We probably wouldn’t have known about it if he [my great-grandson] hadn’t seen it advertised. And I think it’s good for him.

(great–grandmother 5)

##### 3.2.1.4. Shifts in child feeding styles

All caregivers reported shifts in their child feeding styles that affected their child feeding practices. The next generation of children being raised by these caregivers tended to be “pickier eaters” compared to their earlier counterparts. Two different types of shifts in child feeding style were described by participants. 

More than half (11 households) shifted to being more relaxed and indulgent with the second generation. For example, these caregivers reported catering to the food preferences of their grandchildren, and a few struggled not to “spoil” the grandchildren by allowing too many “junk” foods. Another common practice noted was a shift from family meals together at the table to eating around the television or eating separately.

The other five [children] would try anything. He [my great-grandson] don’t want to try [new foods].

(great-grandfather 6)

When we first got him [my grandson], he was very picky, wouldn’t eat a lot, a wide variety of things…. With the first group of kids, we insisted on the table, and when we first got [my grandson], I insisted on the table again. But we’re kind of at that “forget it” age. (laughs) So we do eat together, but it’s in front of the television set.

(grandmother 4)

When the kids grew up, it [eating meals] was always at the table. Now it’s sort of a grab and go.

(grandmother 22)

The second child feeding style shift was characterized as being more involved in feeding. The eight households in this group tended to be more protective of their second group of children, including more carefully monitoring child feeding. This group shifted to more scheduled meals at the table as a family, and they were more nutrition-conscious caregivers.

But I’m more conscious with these kids than I was with my own kids, about nutrition and about everything else. I’m more cautious. What they eat, are they getting what they need? My kids had more snack foods than these kids do…. I have more time to plan meals, to prepare them…. Now we all have dinner together…. Sometimes they [my granddaughters] wouldn’t even eat if one of us didn’t say, “You will eat what’s on your plate.”… And I do see that they eat better than what my kids were allowed to. When my kids were growing up, if they were hungry when they came home from school, they’d grab something, and if they weren’t hungry for dinner, no big deal.

(grandmother 7)

I think [I do] a little better [with feeding my grandson] than with the first group…. But I’ve learned a lot since then. I’ve learned so much more.

(grandmother 17)

##### 3.2.1.5. Sources of child feeding advice

The caregivers stated that over the years, their primary source of child feeding advice was information passed from the previous generation to the caregivers, particularly by their mothers. Additionally, they identified their family doctor or pediatrician as a main source of feeding advice when their first children were born or adopted. Many commented that there was much less emphasis on child feeding by society with their first set of children compared to their second set. When asked if they sought additional sources of nutritional advice for parenting the second time, most described relying primarily on their past parenting experiences, although a few mentioned governmental programs such as the Health Department, Cooperative Extension System, or the Women, Infants, and Children (WIC) program.

My mom… she was the best mom in the world. (laughs) And she was such a good cook. And it [the advice] was just from her…. And I think that you do what your parents did. So you just keep going down that road.

(grandmother 15)

By the time the fourth one [of my children] came along, we moved to Kansas and we didn’t have a pediatrician, but I just fed her the same way as the other ones. And I think it was the doctor that got me going. And [my grandson] was raised the same way.

(grandmother 12)

I was worried about the old group [my children] and tried to do my best, but I didn’t know what all was good at that time…. I knew the basics, coming from Mexico…. When it came to the children later on growing up, when I was babysitting children, the Health Department would provide information on the [Food Guide] Pyramid and nutrition. And that’s where I learned a lot of stuff for my kids, and grandkids, too.

(grandmother 17)

#### 3.2.2. Nutrition-Related Attitudes

Within the nutrition-related attitudes theme, three sub-themes were identified: the caregivers held opinions that nutrition and safe food handling were important; that nutritious food is expensive; and that population-specific nutrition education materials may or may not be helpful either to them or to others in their situation.

##### 3.2.2.1. Nutrition and safe food handling

Attitudes toward nutrition and child feeding shifted, from simply serving meals in order to obtain energy to wanting to serve and eat a balanced diet now. All but two participants expressed at some point during their interviews their belief in the importance of giving this second generation a good start so that they would form life-long nutritious eating habits. Some expressed concerns about the recent rise in childhood obesity and other chronic diseases, such as diabetes, or mentioned having chronic conditions themselves, including diabetes and heart conditions, which made them more aware of the role of nutrition in health. Four caregivers used to view a “good” meal as one that included meat and potatoes, but now have the opinion that incorporating more variety is important, especially including more fruits and vegetables. Three female caregivers mentioned that this “meat and potatoes” attitude came primarily from their husbands.

I’ve had to change the way I think about food. And every once in a while, I get hungry for good ole’ comfort food. It’s hard to give it up. I think in this day and time, if kids grow up eating healthier, they won’t have to go through that.

(grandmother 12)

I think with a child in the house, you’re more aware of what you’re eating and serving. And I think it’s probably going to benefit [my husband] and I in the long run, because we have the desire to be more healthier and to be around for her [my granddaughter] longer.

(grandmother 20)

But trying to give them [my granddaughters] as good a start as possible nutritionally, and learning how to have pop [soft drinks] and stuff like that in moderation. You can have them, but you don’t need them all the time. But we try to encourage fruits and vegetables, and try to get them on a track of good nutrition, so that possibly when they have their own kids, they’ll do the same thing. And maybe that will extend to a healthy lifestyle.

(grandfather 2)

Caregivers believed that it was important to serve less fat, sugar, and salt in their households’ diets. To do this, most thought that looking at food package labels was helpful, while others believed that it was unnecessary for them to read labels because they “already had a good feel for the nutritional value” of the products they buy.

Of course, I look for sugar content. I will not buy anything that has hydrogenated oils. I say I won’t buy—I will occasionally, but not on a regular basis. And if it has, it won’t be up on the second and third [ingredient]. I feel that that’s where a lot of our high cholesterol comes from and our health problems. And I do my best to stay away from that as my main things. Them are the two things I look for on the Nutrition Facts labels…. He [my grandson] knows I have to watch my carbs [carbohydrates] and eat a lot of salads [because of my diabetes]. And he knows that I eat what I’m not supposed to. But he is more aware of food, more than any of my kids was, of the impact it has on your health.

(grandmother 22)

Probably not [helpful to use Nutrition Facts labels], because I pretty much know what’s, what goes together, and your starches and different ones…. And I try to get stuff that’s not so starchy, not so fattening. I do pay attention to the nutrition. Trying to get his [my grandson’s] habits better.

(grandmother 3)

The two main concerns the caregivers expressed about their grandchildren’s eating habits were that the children: were not eating enough food overall; or were eating too much “fast food”, sweets, sports drinks, energy drinks, high fat snacks, and other “junk” foods.

He’s [my grandson] so small and I want him to eat more than he eats, but he doesn’t eat a lot at one time. Now sometimes he’ll get into the junk food and he’ll eat a lot of unnutritious food at one time.

(grandmother 13)

Well, the oldest one [of my grandchildren] wants a lot of junk food. I tell you what! Pizza, pizza, pizza, that kid could live off of pizza.

(grandfather 19)

Regarding attitudes toward safe food handling, the caregivers were of the opinion that storing perishable food in a refrigerator or freezer is important, and also stressed the importance of many other practices. In contrast to their stated opinions that cooking foods properly is important to them, however, only one household reported checking internal temperatures of cooked foods on a regular basis. Rather, most believed that they could adequately tell whether meat was cooked long enough by the way it looked or smelled.

I wash vegetables, that’s for sure! I used to use the foam, but I’m kind of scared. The vegetables that you get around here, like spinach, says it’s washed, but I usually wash it again. And chicken I let soak in salt water. I am too particular sometimes.

(grandmother 1)

‘Cause back when I was growing up and when my kids were small, if you had leftovers, you should let them sit and cool. You shouldn’t put hot stuff in the refrigerator. And I’ve learned since then that you don’t do that…. I always did what my folks did…. I didn’t use to think about it, but I do now.

(grandmother 10)

I make sure that my meat is cooked properly, just make sure it’s done.… I had a meat thermometer, but I don’t use that…. I just know if it’s done if the chicken doesn’t have blood against the bone. It’s just something you pick up when you’ve cooked for so many years.

(grandmother 8)

##### 3.2.2.2. Food and economics

A commonly held food attitude among the caregivers was that eating nutritiously is expensive, but worth it. Most described making monetary sacrifices to eat more healthfully now. Caregivers found ways to eat nourishing foods while minimizing their food expenses.

And economically, now days, in order to buy food that actually fills the kids up, you can’t afford good stuff, because it’s expensive to eat healthy. It’s very, very expensive to eat healthy. And that’s one of the main sacrifices that we’ve had to deal with, is groceries have become so much more expensive. Because we’re paying a lot more attention to what we’re buying.

(grandfather 2)

Especially with the fruit. If it’s grapes and they’re three dollars, I’ll say, “We can’t get it today, [Granddaughter]. We’ll wait until it goes on sale.” And then I’ll go get a can of fruit cocktail or peaches or pears, because I always know that I can get those cheaper.

(grandmother 20)

I’ll look at the fruits and vegetables and see what are the best, price-wise. I try to cut back on my shopping since I can’t do anything about gasoline [prices].

(grandmother 8)

Price influenced what foods were bought and where they shopped for 16 of the households, while the remaining three did not mention price as being a factor in their food shopping decisions. As discussed in the section on caregivers being more nutrition and food safety conscious, some households had more financial resources for food purchases now, and explained that they could eat more healthfully with this generation of children because they had enough money to purchase foods, including more fresh fruits and vegetables. Nevertheless, even most of these caregivers valued keeping their budget in mind while shopping for food.

I haven’t had to be tight… but I’ve had to worry about the expense…. I buy the better eggs…. I have felt like I can buy him [my great-grandson] the better food, nutrition-wise.

(great-grandmother 14)

I don’t skimp on anything. But I don’t buy the most expensive [foods], either. I do keep in mind to buy enough.

(grandmother 7)

These boys [my great-nephews] are probably being fed better than ours were, because just the difference in our jobs and what we could afford to buy.

(great-aunt 16)

##### 3.2.2.3. Population-specific nutrition education materials

Attitudes diverged regarding whether population-specific nutrition education materials would be helpful. However, positive opinions about the usefulness of such materials tailored to skipped–generation(s) caregivers, either to them or to others in their situation, were held by most. Caregivers who were open to the idea of nutrition education stated that the best ways to receive such information would be via: written means, such as brochures and newsletters; videos; having their grandchildren taught about it at school; and organizations, such as local grandparent support groups, Cooperative Extension System offices, and WIC clinics. They did not consider classes to be the best way to share nutrition education. Their suggestions for topics for educational materials focused on nutrition as it relates to children, adolescents, and sports; and on healthful recipes and snack ideas.

I would say more education on what makes you healthy on the outside, ‘cause they’re not going to care at this age [teens] what makes you healthy on the inside.

(grandmother 4)

Maybe not a class but a group that you could ask if there’s a way to do it better or anybody got any suggestions of what would work if it’s not working for me. Just to know that “Sally Smith” down the street is doing the same thing, who’s raising her grandkids, so maybe I am doing something right. Maybe if you’re raising your grandkids, you don’t realize what’s out there that wasn’t available when you were raising your own kids.

(grandmother 11)

I think just a refresher on how many times a week they [children] should get fruits and vegetables, and maybe some alternative foods for snacks that are not high in sugar and are better for them. I think that you would get more people to read something like that than to attend a class. Just to be able to do that when a person had time to sit down and read it or whatever.

(great-aunt 16)

Four of the caregivers had negative attitudes toward nutrition education materials for themselves and others in their situation. They expressed beliefs that most grandparents are going to cook what’s healthy, already have enough information, and would not be willing to change behaviors at this stage in their lives. Older age did not seem to be an indicator of whether the caregivers had negative attitudes.

Mostly grandparents know by now how they’re going to buy food. At my age, there’s not a lot I haven’t learned about food because I’ve been there and done that.

(grandmother 3, age 68 years)

I’ve been involved with my kids, my grandkids, and now my great-grandkids. But things have changed down through the years. Every ten years is different. And I think that if you don’t have some guidelines, you can’t keep up.

(great-grandmother 14, age 80 years)

## 4. Discussion

The participants in our study were married at the same percentage as the national grandparent caregiver population [1]. Our population was a little older, more economically disadvantaged, more rural/frontier, made up of more females and more non-Hispanic whites, and had cared for the grandchildren longer than national and Kansas grandparent caregiver percentages. The caregivers we interviewed were eager to share their experiences, and the information richness of their stories provided great insight into the caregiving aspects of their lives. The circumstances begetting full-time caregiving, the non-nutrition-related challenges, and perceived rewards described by our participants, as reported elsewhere [31], were consistent with the findings of others [2,7,32].

Caregivers in the current study described practices resulting from being more nutrition and food safety conscious with their second group of children. Making more healthful food choices was explicitly related to the presence of the children, at least in some households. One of the largest barriers to eating a nutritious diet for older adults is not having social contact [33], but this problem would not apply to caregivers in skipped-generation(s) households. Our findings of improved nutrition attitudes and practices might not be entirely related to the presence of grandchildren, however, because previous research has shown that older adults tend to make different food choices as they age [34], including consuming higher amounts of fruits and vegetables than their younger counterparts [33]. Also, because many of the caregivers we interviewed did not work outside the home, they were more available to monitor the children’s food choices and environments, which also might have led to more healthful food and activity choices [35]. 

The caregivers in our study highlighted an on-the-go lifestyle and children’s use of more electronics as adversely affecting the children. Nutrition challenges resulting from an on-the-go lifestyle, such as occasions of increased consumption of packaged foods and “fast food” described by some of our participants, have been reported in a previous study of grandparent caregivers [17]. The caregivers in our study were rightly concerned about the increased time spent using electronics, and thus more sedentary activity, for their grandchildren, because eight of the households in our study reported in excess of the American Academy of Pediatrics’ recommendation of no more than one to two hours of quality television programming per day [36] and because of the previously-reported positive correlation between screen time and childhood obesity [37]. Likewise, our caregivers’ perceptions of the influence of televised food advertisements on their grandchildren’s food requests are supported by previous research [38]. 

Our finding that caregivers shifted their child feeding style to be either more controlling or more relaxed might have important health implications for the children. Research points to the importance of balanced familial control of child food choices. Children who grow up in households in which food choices are too controlled or restrictive tend to overeat the restricted foods in excess, even when they are not hungry, when able to do so [23,39]. Similarly, children who grow up in households in which food choice is unregulated tend to be more likely to consume large amounts of soft drinks and sweets, and fewer vegetables [15]. 

Skipped-generation(s) caregivers in the current study described relying mostly on information from their mothers, *i.e.*, “tradition”, their physicians, and their past parenting experiences for nutritional advice when they were raising their second set of children. In contrast, primary sources of child feeding advice for new parents may also be tradition, family, or a doctor [40] or they may actively seek current infant and child feeding recommendations [41]. Our caregivers may have believed that they had adequate infant and child feeding information, since they had already raised a child(ren), not realizing that while some nutrition-related “expert recommendations” have remained constant over time, others have changed dramatically. In addition, although many stated that they were relying on past parenting experiences, they also described practicing newer behaviors, such as checking Nutrition Facts labels. The seeming discrepancy can be explained if traditional sources of information are seen as a way of conveying either old or new information while using informal channels of communication, for example, via television, magazine stories, and social networks. 

With their newest generation of children, our participants reported that their nutrition-related attitudes had improved, along with their practices. For example, the higher value that they now place on eating healthfully led them to change their food selection behaviors, including serving a wider variety of foods and, especially, more fruits and vegetables. Remarkably, many described cooking more foods from “scratch” for their grandchildren, despite the increased numbers of packaged and convenience foods in grocery stores now. Our findings that caregivers found the fat, sugar, and sodium information on the Nutrition Facts labels to be the most helpful agree with findings from the 2007 Food and Health Survey of adults of all ages [42]. Compared to younger consumers, older adults are less likely to change their shopping behaviors based on Nutrition Facts labels; however, nutrition labels have a larger effect on older adults’ nutrition attitudes toward the products if they know more about nutrition and understand the labels [43]. Thus, as their nutrition knowledge increases, and Nutrition Facts labels become more familiar to the older population, these labels might begin to affect older consumers’ purchasing practices, as implicated in our findings. 

Our participants also described having both improved attitudes and practices towards handling food safely, compared to their opinions on this issue with their first set of children. For example, they now believe that refrigerating leftovers immediately after meals, instead of letting them cool on the counter, is important. From a historical perspective, “expert recommendations” for consumer practices have changed drastically over the caregivers’ lifetimes, as a result of many factors, and these likely have helped increase the caregivers’ awareness of the importance, and relevance, of home food safety practices. For instance, the food safety attitudes of older adults may be influenced by whether they subscribe to the “germ theory” of disease. Since the theory had not become popular in the U.S. until as late as 1920 [44], many of our caregivers likely grew up without benefit of such microbiological information. A second example of dramatic change related to safe food handling practices that these caregivers have experienced is that the oldest participants in this study were at least 10 years old before any kind of electrical service began in Kansas [45]. Because the attitudes of older adults toward food safety strongly correlate with their prevention practices [46,47], our study’s findings regarding attitudes about which food handling practices were the most important have implications for the practices of the caregivers, and can inform future food safety education. For example, the commonly held opinion that meat can be cooked safely without checking its internal temperature could be used as the basis for population-specific education. 

We found that economic issues, and attitudes that healthful foods are expensive, strongly influenced our caregivers’ food shopping behaviors. This finding is likely linked to the high (37 percent) number of the households in our study who were receiving monetary assistance that could help purchase food. In contrast, many of the monetary and food assistance programs that can assist these low-income households, such as medical assistance, emergency food providers, free and reduced-price school meal programs, the WIC program, and the Supplemental Nutrition Assistance Program, may not be familiar to eligible skipped-generation(s) caregivers [48]. 

Our participants, who had fewer financial resources than the national average, yet who valued good nutrition, described buying less costly healthful foods, such as canned fruit instead of fresh. Attitudes that healthful foods are expensive have been reported for other caregiver studies [12,17]. Nutrition educators are advised to be mindful of both real and perceived financial barriers to making healthful dietary choices. In particular, because the attitude that “good food is expensive” is a recurring theme in older adult research, including international caregiver research [49], educators should stress the viability of making meals that are low in cost yet high in health benefits with this population. 

Nutrition education should result in behavior change, and by design, should be tailored to the audience it is intended for based on their assessed needs and interests [50]. Caregivers in this study discussed wanting educational materials that focused on nutrition as it relates to children, adolescents, and sports; and on healthful recipes and snack ideas. In addition, based on their reported practices and attitudes compared to current expert nutrition recommendations, nutrition education for this population should include quick and inexpensive healthful meals that are low in fat, sugar, and salt; healthful “fast food” and packaged food options; the importance of checking the internal temperatures of meat when cooking; infant and child feeding; ways to feed “picky eaters”; benefits of eating together as a family; tips to help children learn to cook and to limit their sedentary time; and intergenerational gardening and cooking. 

Cooking and eating inexpensive yet healthful meals as a family, along with being physically active, such as gardening together, would offer skipped-generation(s) household members daily opportunities to benefit in some areas where they may be experiencing difficulties. For example, eating and gardening with children are associated with frequent and uncontrived chances for relaxed communication and emotional connections with each other; a boost in decision-making skills, confidence and self-esteem; improved math, science, and language skills and general academic achievement of children; decreased likelihood of risky behaviors by the younger generation; and overall more positive familial and other social relationships [51,52]. Thus, providing caregivers in skipped-generation(s) households with comprehensive nutrition education would be expected to lead them to be healthier older adults and more effective second-time-around parents, and at the same time, help to ensure a healthier future for the children being cared for by this group.

The caregivers in this study who were open to the idea of nutrition education were of the opinion that the best ways to receive information were via: written means, such as brochures and newsletters; videos; having their grandchildren taught about it at school; and organizations they trust, such as those we recruited from (local grandparent support groups and Cooperative Extension System offices) and WIC clinics. Published literature confirms that print and video sources were also preferred by other groups of older adults [53], including grandparents raising grandchildren [32], with printed newsletters being inexpensive yet effective. Support groups as one of the preferred ways to receive informational help and to relieve stress is congruent with findings from other skipped-generation caregiver studies [6,12,54]. 

Our research focused exclusively on skipped-generation(s) caregivers providing full-time surrogate care to dependent children in the absence of their parents. The results, however, also have implications for providing nutrition education to older adults who either regularly or occasionally provide supplemental care to the younger generation. For instance, many grandparents provide day care for their grandchildren, and, as such, are responsible for providing most of the meals and snacks eaten by the infants, children and teens under their supervision. The same may be true for older adults who make their living as paid day care providers.

## 5. Limitations

This study was designed to explore and describe a broad scope of nutrition-related practices and attitudes, which limited the study depth into any one area. The sample in this study was not demographically representative of the population of grandparent caregivers as a whole, in racial/ethnic groups, gender, or residence. Additionally, a generally positive response bias is associated with self–selected community samples of middle-aged and older adults [5]. While we used no objective measures for participants’ truthfulness, they seemed to speak from their hearts and to believe strongly in what they stated; we have no reason to think that they falsified any descriptions shared with the interviewer. We did not interview enough people to learn about all situations, practices, and attitudes; a point of data “saturation” likely was not reached. Our findings should be considered preliminary work leading to more research in this area of study, and not be generalized. 

## 6. Conclusions

Nutrition is just one of the often-complicated challenges experienced by members of skipped–generation(s) households, although it is an important variable in health. This study provides beginning insight into the practices and attitudes of skipped-generation(s) kinship caregivers in their role as nutrition providers for the dependent children in their care. 

Based on content analysis of personal interviews, caregivers described being more nutrition and food safety conscious compared to when they were parenting the first time. Five changes in their practices included: serving a more nutritious variety of foods, reading Nutrition Facts labels, doing more cooking, storing foods properly, and keeping food preparation areas clean. The caregivers mentioned new challenges to their dependent children eating nutritionally-balanced meals because of shifts toward an on-the-go lifestyle. An increased use of electronics by the children increased their sedentary activity and family purchases of advertised foods. Many caregivers noted shifts in their child feeding styles, which influenced their child feeding practices. The two types of shifts were being more relaxed and indulgent with the second generation, or being more involved in feeding now. Our participants credited their child feeding knowledge primarily to information from their mothers, a doctor, or, with the newest generation, to their past experiences. Caregivers had an improved attitude toward nutrition and safe food handling, compared to the regard with which they held these practices with their first generation of children. They perceived economic issues as a challenge to selecting healthful diets. Participants recommended that if nutrition education materials were developed for this population, they should be distributed primarily using printed or video materials and wanted to receive information through organizations they trusted. 

Based on the results, in juxtaposition with current expert nutrition recommendations, the authors conclude that this population could benefit from education on nutrition as it relates to infant, child, adolescent, and sports nutrition; feeding “picky eaters”; healthful recipes, “fast foods” and packaged foods; quick, inexpensive meals and snacks low in fat, sugar, and salt; limiting sedentary time; family meals; using food thermometers; and intergenerational gardening and cooking. The findings have implications for nutrition educators and other health professionals, who can help older skipped–generation(s) caregivers be healthier older adults and more effective parents, and help ensure a healthier future for the dependent children in their care.
